# To target or not to target? The role of DNA and histone methylation in bacterial infections

**DOI:** 10.1080/15592294.2023.2242689

**Published:** 2023-09-20

**Authors:** Magdalena Barbachowska, Paola B. Arimondo

**Affiliations:** a Institut Pasteur, Université Paris Cité, CNRS UMR n°3523 Chem4Life, Epigenetic Chemical Biology, Department of Structural Biology and Chemistry, Paris, France; b Universite Paris Cité, Ecole Doctorale MTCI, Paris, France; c Institut Pasteur, Pasteur- Paris University (PPU)- Oxford International Doctoral Program, Paris, France

**Keywords:** Bacterial infection, DNA and histone methylation, chemical targeting

## Abstract

Epigenetics describes chemical modifications of the genome that do not alter DNA sequence but participate in the regulation of gene expression and cellular processes such as proliferation, division, and differentiation of eukaryotic cell. Disruption of the epigenome pattern in a human cell is associated with different diseases, including infectious diseases. During infection pathogens induce epigenetic modifications in the host cell. This can occur by controlling expression of genes involved in immune response. That enables bacterial survival and replication within the host and evasion of the immune response. Methylation is an example of epigenetic modification that occurs on DNA and histones. Reasoning that DNA and histone methylation of human host cells plays a crucial role during pathogenesis, these modifications are promising targets for the development of alternative treatment strategies in infectious diseases. Here, we discuss the role of DNA and histone methyltransferases in human host cell upon bacterial infections. We further hypothesize that compounds targeting methyltransferases are tools to study epigenetics in the context of host-pathogen interactions and can open new avenues for the treatment of bacterial infections.

## Introduction

Bacterial infections affect DNA and histone methylation pattern in the host as a strategy for bacteria to improve pathogenesis and escape the immune response to infection. This review focuses on DNA and histone methylation in human cells in response to bacterial infections. In particular, it underlines the impact of the bacterial infection through methylation of the host genome on its immune response. Finally, we discuss that epidrugs that target these aberrant DNA and histone methylation patterns in host cells can constitute an alternative therapy to fight infectious diseases.

## DNA and histone methylation in mammals

**Figure 1.**
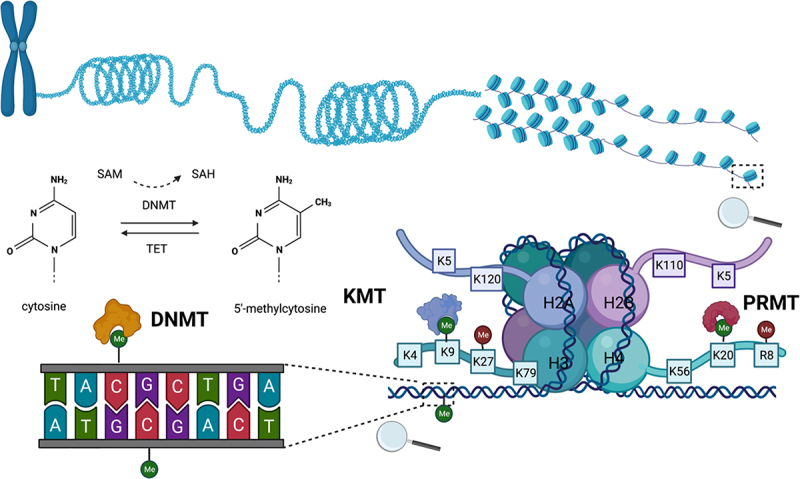
DNA and histone methylation in mammals. The DNA molecule is wrapped around histone octamers (eight histones consisting of dimers: H2A, H2B, H3, H4) to form the nucleosome, further compacted into chromatin fibres, the unit of chromatin that forms chromosomes. In mammals, DNA is mainly methylated at cytosine position 5 by the DNMTs (DNA methyltransferases). Whereas histones are methylated at lysine (K) and arginine (R) residues by KMTs (lysine methyltransferases) and PRMTs (protein arginine methyltransferases), respectively. The methylation reaction is catalysed by the methyltransferases using SAM (*S*-adenosyl-L-methionine) as a cofactor and donor of methyl group and release of SAH (*S*-adenosyl-L-homocysteine) and reversed by the demethylases. Created with BioRender (biorender.com).

The DNA double helix molecule encodes the genetic information for the function, growth, development and reproduction of an organism [1]. A human genome consists of more than 25,000 genes and a diploid genome is around 6,400 mega base pairs (Mbp) in size packed into a nucleus of a cell of about 10 microns in diameter [2,3]. To fit into the nuclei, the DNA is highly compacted. The first level of this compaction consists of the nucleosomes, a sequence of 147 bp of DNA wrapped around a histone octamer (eight histone proteins consisting of two copies of the dimer H2A- H2B and two copies of the dimer H3- H4) [4] (Figure 1). Histone proteins are abundant in positively charged arginine and lysine amino acids that interact with negatively charged DNA. This tight interaction enables dense packaging of the DNA molecule into the cell nuclei. The nucleosome is the basic unit of chromatin that is dynamic and may form open (euchromatin) and condensed structure (heterochromatin) [5]. Transcription factors may bind to promoter regions and enhancers in euchromatin but not in the heterochromatin state, in which transcription is inactive.

Epigenetics describes modifications in the genome that control gene expression by modulating chromatin compaction without altering the DNA sequence. The main epigenetic modifiers are *writers, readers* and *erasers*, proteins that add, bind to, and remove the epigenetic modifications [6]. In mammals, widely studied chemical modifications are methylation of DNA and methylation, acetylation, ubiquitination, phosphorylation, and crotonylation of histones. The main function of epigenetic modification is regulation of gene expression. An epigenetic modification impacts chromatin structure and the accessibility of the transcription factors and enhancers to bind to DNA as shown by novel state-of-the-art sequencing techniques [7,8]. Moreover, epigenetic modifications, also named marks, play an important role in the formation of protein complexes and machineries during replication and transcription [9].

DNA methylation is implicated in chromatin remodelling [10] and its effect on gene transcription depends on the DNA region that is methylated. DNA methylation most commonly correlates with repression of transcription when occurs in promoters [11], while activation of tissue-specific gene transcription is observed for example in gene bodies [12]. Histone methylation occurs on arginine (R) and lysine (K) residues, mostly on histone tails [13]. The effect of histone methylation on transcription depends on the residue and the methylation state mono-, di-, trimethylation for lysines (abbreviated as Kme1, Kme2, Kme3) and mono- and symmetric or asymmetric dimethylation for arginines (Rme1, Rme2s, Rme2a) [14,15]. Selected methylation marks are presented in Table 1 with examples of the corresponding methyltransferases and the effect of the methylation on transcription (Table 1). Depending on which part of the genome the modification occurs and the level of methylation, it can induce gene repression or activation as cited in Table 1.

**Table 1.** DNA and histone modifications and methyltransferases in mammals.

Modification	Methyltransferase		Transcription regulation	References
DNA 5mCpG	DNMT1, DNMT3A, DNMT3B		Repression/activation	[16–18]
H3K79me1/2/3	DOT1L		Activation	[19]
H3K36me2/3	NSD1, NSD2		Activation/repression	[20]
H3K36me3	SETD2		Activation	[21]
H3K27me1/2/3	G9a/GLP, EZH1, EZH2		Repression	[22,23]
H3K9me1	PRDM3, PRDM16, G9a/GLP		Activation	[24,25]
H3K9me2/3	G9a/GLP, SUV39H1 SUV39H2, SETDB1, SETDB2		Repression	[25–28]
H3K4me1/2/3	SET1A, SET1B, MLL1, MLL2, MLL3, MLL4, MLL5, ASH1L, SMYD3		Activation	[29–32]
H3R17me1/2	CARM1		Activation	[33]
H3R2me1	PRMT1, PRMT3, PRMT5, PRMT6, PRMT7, CARM1		Activation	[34]
H3R2me2a	PRMT1, PRMT3, PRMT6		Repression	[35–37]
H3R2me2s	PRMT5, PRMT7		Activation	[38]
H4K20me1	SET8	Activation		[39]
H4K20me3	SUV420H1, SUV420H2	Repression		[40–42]

### Mechanism of DNA and histone methylation

The methylation mechanism is based on the transfer of a methyl group from the donor (the co-factor *S*-adenosyl-L-methionine, SAM) to the substrate (the cytosine in DNA or arginine or lysine of histones) with release of *S*-adenosyl-L-homocysteine (SAH) as a byproduct [43] (Figure 1). The reaction is catalysed by the DNA methyltransferases DNMT or the histone methyltransferases (HMTs), the *writer* proteins. The histone lysine or arginine residues’ methylation reaction is catalysed by lysine methyltransferases (KMTs) or protein arginine methyltransferases (PRMTs), respectively [13,44]. The majority of the KMTs bear the SET domain and therefore are grouped into SET-domain protein family. The SET-domain is a conserved sequence motif that forms a catalytic active site for lysine methylation [45]. The exception is the DOT1L family that does not have SET-domain but another conservative sequence motif [46]. Whereas, PRMTs are distinguished by a conserved catalytic core region of about 310 amino acids [47].

In mammals, DNA methylation occurs mostly on position 5 of cytosine (5meC) at CpG sites that are regrouped in CpG islands (CpG-rich regions) (Figure 1). There are three different DNA methyltransferases: DNMT3A, DNMT3B, and DNMT1. DNMT3A and DNMT3B catalyse *de novo* DNA methylation and are active during early stages of the development in embryogenesis and gametogenesis [43]. Whereas, DNMT1 maintains methylation marks during cell division.

The methylation reaction is reversed by enzymes called demethylases, the *erasers* [44,48]. The DNA demethylation process undergoes several oxidation steps catalysed by the TET enzymes (Ten-Eleven-Translocation dioxygenases) [49,50]. Whereas, histone methylation is reversed by histone demethylases. The proteins called *readers* recognize and bind to the methyl groups (on DNA or histones) [51,52]. Many corresponding *reader* proteins are described to recruit protein complexes during transcription and triggering signalling cascades involved in gene regulation [53–55]. The cooperation of writers, erasers and readers ameliorates the dynamics and complexity of the process of gene expression.

Epigenetics plays an important role in regulation of cellular processes and cell functions. Consequently, disruption of the methylation pattern is found in many pathologies including cancer, neurodegenerative diseases, ageing, and infectious diseases [56].

In the following part we will focus on the role of methylation in bacterial infectious diseases.

## Role of host DNA and histone methylation in infectious diseases

In bacteria, DNA methylation marks include N^6^-methyladenine, N^4^-methylcytosine, and 5-methylcytosine (for review [57]), the most common modification being N^6^-methyladenine involved in the defence against pathogens, mostly bacteriophages [58]. Bacteria distinguish exogenic DNA from its own by restriction enzymes (endonucleases) that recognize specific unmethylated sequences of foreign nucleic acids. DNA adenine methyltransferase (DAM) and its homologs are present in various bacteria [58]. DAM methyltransferase is also present in some of the bacteriophages, for example *Escherichia* phage P1 (Bacteriophage P1) [59]. Although, there is no histone methylation in bacteria there are histone-like proteins that can be post-translationally modified [60]. Here we focus on DNA and histone methylation induced by the bacteria in human cells.

**Figure 2.**
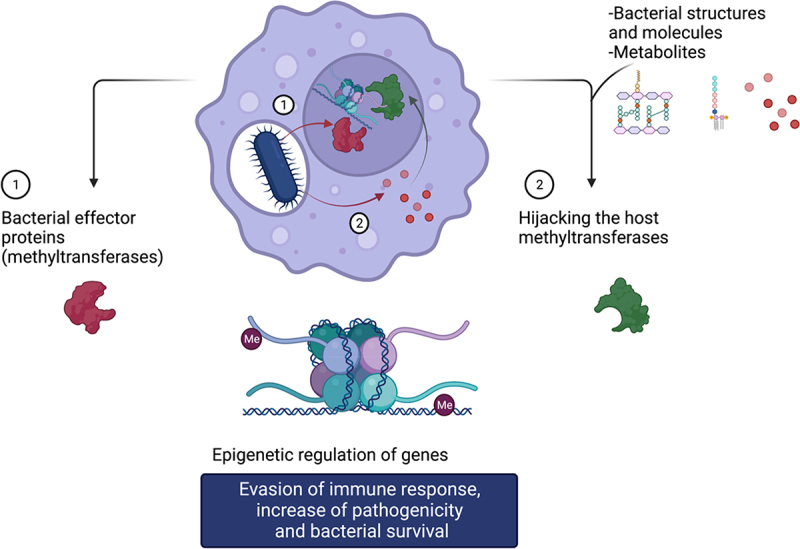
Bacteria infection induce epigenetic changes in the host. Bacteria modify the genome of the host through 1) release of bacterial effector proteins (such as methyltransferases-like proteins) that modify the host genome or 2) stimulation of the host cell environment through release of bacterial molecules or stimulation of signalling pathways by bacterial structures to hijack epigenetic modifiers including DNA and histone methyltransferases. Created with BioRender (biorender.com).

Epigenetic modifications including DNA and histone methylation are implicated in various mechanisms that increase pathogenicity, virulence, and support immune response evasion during infection [61]. Bacteria can modify the host epigenome either by secretion of effector proteins that mimic host methyltransferases or by hijacking and regulating functions of host methyltransferases through production of microbial molecules, induction of the changes in the microenvironment and metabolic switch (Figure 2). The epigenetic regulation of the host genes is clinically relevant to understand mechanisms of infection and opens to novel targets for treating bacterial infections.

## How bacterial infection modulates the epigenome?

Bacteria modulate the host epigenome by (1) either directly inducing methylation changes in the host epigenome through effector proteins that catalyse the methylation or (2) by hijacking the host methyltransferases (Figure 2). The two mechanisms are described through examples.

### Bacterial effector proteins induce epigenetic methylation changes in the host

As a result of evolutionary mimicry, bacteria secrete effector proteins that resemble methyltransferases [61,62]. Nucleomodulins are bacterial effector proteins that are secreted into the host cell upon infection and translocate into the nucleus through nuclear localization signal (NLS). In the nucleus, nucleomodulins modify epigenetic pattern of the host genome, alter microenvironment and activate or inhibit signalling pathways implicated in the regulation of gene expression [61]. For instance, bacteria can target the NF-κβ signalling pathway with observed decrease in pro-inflammatory cytokines (e.g., IL-6 TNF-α) resulting in repression of genes associated with immune response [63]. Therefore, bacteria evade the immune response, escape clearance from the cell and persist within the host cell to proliferate and survive during infection through epigenetic reprogramming of host-pathogen interactions [64]. Nucleomodulins are secreted by a variety of bacteria, both Gram-positive and Gram-negative, both extracellular and intracellular pathogens, including *Legionella pneumophila, Burkholderia thailandensis, Chlamydia trachomatis*, *Chlamydophila pneumoniae, Bacillus anthracis, Mycobacterium tuberculosis, Mycoplasma hyorhinis*, *Klebsiella pneumoniae*, *Salmonella enterica* and archaea *Methanosarcina mazei*. Bacterial methyltransferases target DNA regions of different genes, histones (H1, H3, H4) and non-histone proteins. Table 2 gathers selected effector proteins that modulate the host epigenome and regulate gene expression in favour of an efficient pathogenesis, therefore playing an important role during infection.

**Table 2.** Examples of bacterial effector proteins that methylate host substrates.

Pathogen	Bacterial effector protein	Mammalian methylation target	Impact on the host	Reference
L. pneumophila	RomA/LegAS4	H3K14, non-histone proteins	Repress genes involved in innate immune response (e.g., IL6, TNFA)	[63,65,66]
B. thailandensis	BtSET	H3K4 methylation in rDNA	Induction of rDNA transcription and promotion of bacterial intracellular replication	[66]
C. trachomatis	Nue	H2B, H3 and H4	Chromatin remodeling	[67]
B. anthracis	BaSET	H1	Repression of the NF-κβ pathway	[68]
M. tuberculosis	Rv1988	H3R42	Repression of genes involved in host defense	[69]
M. hyorhinis	CG- and GATC-specific DNMT	CG- and GATC-specific DNA	Activation of oncogenes and promotion of cell proliferation	[70]
K. pneumoniae	HsdM	DNA methylation *in vitro*	Unknown	[71]
S. enterica Typhi	upregulation of KDM6B	Decrease of H3K27me3	Activation of DAAM1, PPARδ, CSNK1D	[72]
M. mazei	Gö1-SET	MC1-α at lysine 37	Unknown	[73]

Human gastrointestinal tract is an environmental niche for both commensal and pathogenic bacteria [74]. A Gram-negative bacteria *Salmonella enterica* serotype Typhi (*S. typhi*) is a pathogen found in human gut microbiota when infected food is ingested. Upon infection, the *S. typhi* modifies the epigenome of human gut cells to evade immune response and support chronic infection [72,75]. As shown in Table 2, *S. typhi* reprograms host-pathogen interactions through the functional *Salmonella Pathogenicity Island I (SPI1) loci* that leads to upregulation of histone demethylase KDM6B and abolishment of H3K27me3 in human colonic epithelial cells and *in vivo* [72]. Consequently, decrease in H3K27me3 activates expression of genes through WNT signalling pathway upregulating *DAAM1, PPARδ, CSNK1D*, involved in cytoskeletal reorganization, fatty acid oxidation and casein phosphorylation. The epigenetic changes induced by *S. typhi* shift polarization of macrophages into M2 macrophages to evade immune response and survive within the host cell. These changes are cell-type-specific [75].

### Bacteria hijack methyltransferases of the host

In parallel, certain components of the bacteria and produced molecules can also affect the action of the host methyltransferase and induce methylation changes that favour the bacterial infection. These components are either recognized by host receptors that trigger signalling pathways or are delivered to the host cells.

#### Bacterial structures and molecules are recognized by the receptors on the host cells

**Figure 3.**
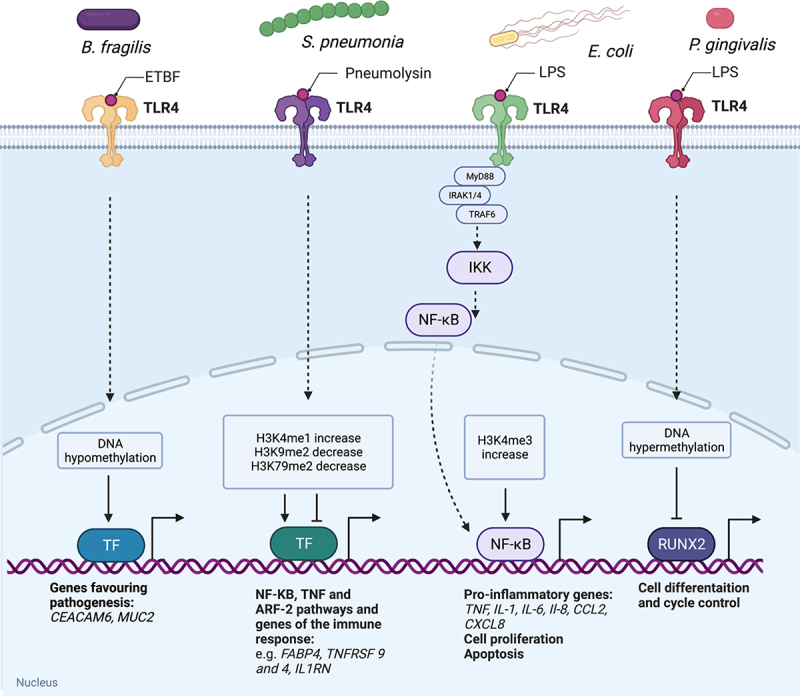
Bacteria regulate gene expression through epigenetic control via TLR signalling. Bacteria-host interaction activates TLR signalling that triggers gene expression signalling pathways. Epigenetic modifications (such as DNA and histone methylation) affect the binding of transcription factors (TF) to promoters. Bacteria release ligands that activate TLR4, for example *B. fragilis* (ETBF), *S. pneumonia* (Pneumolysin), *E. coli* (LPS), *P. gingivalis* (LPS)), and introduce changes in the host epigenome. Depending on the DNA or histone mark, different transcription factors are affected, such as NF-κβ, RUNX2 and transcription factors favouring efficient pathogenesis (genes of immune response, pro-inflammatory cytokines and chemokines, adhesion molecules, cell proliferation, cell differentiation). Created with BioRender (biorender.com).

During infection, bacteria secrete several molecules and structures, among them virulence factors such as toxins, that can induce epigenetic modifications in the host (Figure 3). These are recognized by the host cell through receptor recognition. The receptor-dependent host-pathogen recognition activates intracellular signalling in the infected cell that results in hijacking of the host methyltransferase and ultimately triggers modifications of the host epigenome.

Toxin from *Bacteroides fragilis* (called Enterotoxigenic Bacteroides fragilis (ETBF)-secreted *Bacteroides fragilis* toxin (BFT)), is linked to inked to the development and progression of colorectal cancer caused by epigenetic changes in the host [76]. Based on the transcriptomic and methylome data combined with chromatin accessibility assays, the authors observed dynamic changes in chromatin accessibility and DNA hypomethylation of promoter regions of several transcription factors, genes involved in bacterial pathogenesis (e.g., *CEACAM6, MUC2*) and development and progression of colorectal cancer (e.g., *FOSL1, JUN* and *JDP*) [76]. Infection by *Helicobacter pylori* (*H. pylori*) is linked to development and progression of gastric cancer. *H. pylori-*infected cells show hypermethylation in the promoter regions of various transcription factors, including upstream stimulatory factors 1 (*USF1*) and *USF2*, involved in gene regulation related to immune response, cell cycle and cell proliferation, consequently leading to downregulation of *USF1* and *USF2* [77–79]. The treatment with 5’-azacytidine (5azaC), inhibitor of DNMT, abrogated hypomethylation and re-established expression of USF1 and USF2 *in vivo* [80]. Moreover, *H. pylori* inhibits telomerase activity by hypermethylation of the promoter of Telomerase Reverse Transcriptase (*TERT*) in *TERT* human gastric epithelial cells and murine gastric mucosa in infected mice. The expression of TERT is recovered upon treatment with 5azaC *in vivo* [49].

Some bacterial structures and molecules are pathogen-associated molecular patterns (PAMPs) that are recognized by receptors, *e.g.*, toll-like receptors (TLR) and NOD-like receptors (NLR) on the host cell [81]. The PAMPs-host cell receptor recognition can activate a cascade of signalling pathways, release of cytokines and metabolites that change the microenvironment of the infected cell, shifts metabolism, induces oxidative stress and redirects cell to necrosis through changes in the methylation pattern of the host cell. Ultimately, these changes suppress immune response to infection by evading clearance of the bacteria from the cell and allow bacterial replication and survival within the host [81]. Pneumolysin is a virulence factor released by *Streptococcus pneumoniae* recognized by the Toll-like receptor 4 (TLR4) on the host cell and triggers immune response via nuclear factor-κβ (NF-κβ) signalling pathway through epigenetic regulation [82]. Cole *et al* studied the epigenome of the human monocyte derived macrophages (MDM) exposed to pneumolysin during *S. pneumoniae* infection. The study evaluated the relative abundance of histone marks in MDM cells challenged with *S. pneumoniae* expressing pneumolysin compared to *S. pneumoniae* mutants not expressing pneumolysin showing an increase in the relative abundance of histone marks H3K4me1, H4K16ac and a decrease in H3K9me2 and H3K79me2 in MDM challenged with pneumolysin. These changes in histone marks confirm the manipulation of the host epigenome by bacteria.

The main component of Gram-negative bacterial cell wall is lipopolysaccharide (LPS). It has been shown that LPS can stimulate epigenetic changes in many cell types, including macrophages of central nervous system, monocytes, keratinocytes, fibroblasts, and endothelial cells. During infection, LPS binds to TLR4 on the host cell triggering a cascade of signalling pathways that activate transcription factors such as NF-κβ and interferon-regulatory factors (IRFs) implicated in the expression of genes coding for pro-inflammatory cytokines and chemokines such as cyclooxygenase-2 (COX-2), interleukin-6 (IL-6), IL-1ß, tumour necrosis factor-α (TNF-α), and IL-12. It also activates phosphatidylinositol 3- kinase (PI3K)/Akt and mitogen-activated protein kinase (MAPK) signalling pathways [83]. During activation of NF-κβ signalling pathway, the repressive mark H4K20me3 is decreased, which represses expression of TLR4 in macrophages important for recognition of LPS during infection-activated immune response [84]. Stimulation of macrophages with LPS activates TLR-mediated signalling and regulates expression of DNA and histone methyltransferases. Mouse macrophages of central nervous system stimulated with LPS induced expression of IRF1, IRF7, and IRF9 and upregulation of DNMT3L, histone methyltransferase SETDB2, and histone demethylases KDM4A through TLR4 activation [85]. This suggests that epigenetic regulation is involved during immune response to LPS stimulation in the mouse-derived macrophage.

Other examples of the link between LPS and the epigenetic modulators come from bacteria *Porphyromonas gingivalis* involved in *P. gingivalis*-associated periodontitis. Stimulation of human HaCaT keratinocytes with LPS from *P. gingivalis* resulted in the downregulation of DNMT1 and DNMT3a and H3K27me3 demethylase JMJD3 [86]. In fibroblasts derived from human periodontal ligament (HPDL), *P. gingivalis* LPS-stimulation increased DNMT1 expression with, in particular, DNA hypermethylation of RUNt-related transcription factor 2 (RUNX2), involved in cell cycle regulation. Treatment with DNMT inhibitor, 5-aza-2’-deoxycytidine (5azadC) reversed DNA hypermethylation and diminished DNMT1 expression.

Stimulation of macrophages with LPS from *E. coli* caused the increase in H3K4me3, which activated the NF-κβ signalling pathway mediated and expression of pro-inflammatory genes Nos2 and IL6 [87]. LPS triggered the expression of enzymes involved in methylation such as PRMT4 (CARM1), PRMT6, and SET7/9 in kidney cells. These methyltransferases deregulated global histone arginine and lysine methylation as well as DNA methylation and induced pro-inflammatory response [88].

#### Microvesicles deliver bacterial structures and molecules to the host

The bacterial structures and molecules can also be delivered into the infected cell via microvesicles (MV), small particles of around 50–100 nanometre in diameter shredded from the layers of outer surface of the bacterial cell. MV carries different components of the bacterial cell including components of the periplasm (LPS, peptidoglycan, phospholipids) as well as molecules found in the cytoplasm (nucleic acids, proteins, enzymes, metabolites and toxins) [89]. In *L. pneumophila* infection, bacterial MV are carriers of miRNAs that target host immune genes [90]. The extracellular particles play an important role in delivery and transport of different compounds and are implicated in cell signalling. For instance, Vdovikova *et al* showed that MV isolated from pathogenic bacteria *Vibrio cholerae* and non-pathogenic bacteria *E. coli* induced an increase in H3K4me3 at transcription start sites (TSS) region changing global gene expression of 465 and 738 genes in colorectal cancer cells, respectively [91]. Only MVs from the pathogen *V. cholerae* activated genes associated with endothelial differentiation, contributing to cancer development and progression. Another study on the role of MV in infection investigated the effect of pathogen *P. aeruginosa* on lung macrophages isolated from patients diagnosed with cystic fibrosis (CF). Armstrong *et al* observed a decrease in DNA methylation of the *CFB* gene proposing an epigenetic mechanism of MHC suppression in infected macrophages of CF patients [92]. The DNA hypomethylation induced the suppression of eleven different MHC class II molecules that are important for antigen presentation during infection and activation of immune response. Simultaneously, the study showed a disrupted microenvironment in the infected macrophages, with an increase in pro-inflammatory molecules (IL1β, IL8, CXCL1), anti-inflammatory genes (*IL6* and *IL10*), leukocytes (both T and B cells) and chemoattractants (CCL18 and CCL23). Moreover, MV from *P. aeruginosa* triggered alterations in DNA methylation affecting immune response in human lung macrophages. Consequently, bacterial MV induced DNA hypomethylation in enhancer, DNase hypersensitive regions and gene body regions including *NFKB1, CREB5, BCL2, IL1B* and *IL6*. These observations suggest the involvement of DNA methylation in regulation of NF-κβ pathway during innate immune response [93].

Another example of bacterial MVs affecting epigenetic pattern in the host are MVs from *Bacteroides thetaiotaomicron*. Murine bone marrow-derived monocytes treated with MVs from *B. thetaiotaomicron* showed increased levels of increase levels of H3K4me1, together with IL-10 and decreased levels of TNF- α [94]. Further studies are summarized in [95,96].

## Bacterial infection induces epigenetic modifications implicated in trained immunity

The epigenetic modifications have an impact on the fate of the immune cells to become either effector or memory T cell. The changes in the methylation pattern induced by bacterial infection regulate genes involved in the development of trained immunity. For instance, an *in vivo* study of Laval *et al* showed that haematopoietic stem cells (HSCs) derived from mice exposed to LPS from *P. aeruginosa* induced long-term epigenetic alteration of chromatin accessibility in the C/EBPb pathway, induced by direct LPS-TLR4 recognition [97]. Moorlag *et al* showed that mice exposed to β-glucan showed an increase in H3K27ac, K3K4me3 in the *IL1* genes and a decrease in H3K9me3 at *IL1B* gene, when re-exposed to *Mycobacterium tuberculosis* (*M. tuberculosis*) [98]. Monocytes exposed to bacillus Calmette-Guérin (BCG) vaccine against tuberculosis are reprogrammed to trained immunity response via IL-1β- induced epigenetic regulation in promoter regions of *TNFA*, *IL6* and *IL1B* caused by the increase of H3K4me3 and a decrease in H3K9me3 [99].

Moreover, murine Natural killer (NK) cells stimulated with LPS for 13 days and re-exposed to LPS on day 14 differentiated into memory NK cells *in vivo*. This was mediated by an increment in H3K4me1 within enhancers at the *ifng* locus and increase in INF- γ in murine memory-like NK cells [100]. The treatment with sinefungin, a non-specific inhibitor of methyltransferase, reversed methylation of H3K4me1 and disabled differentiation of NK cells into memory cells [100,101]. Thus, histone methylation is involved in the immune response to bacterial infection.

The abovementioned observations emphasize the importance of understanding the mechanisms of epigenetic modifications in infection, as it could open to the discovery of novel strategies in treatment of infectious diseases. Thus, chemical inhibitors of these modifications could be useful tools to investigate the epigenetic modifications in host cells upon bacterial infections and, depending on their properties, become the starting point for the development of novel drugs.

## Perspectives of chemically targeting epigenetics in bacterial infections

**Figure 4.**
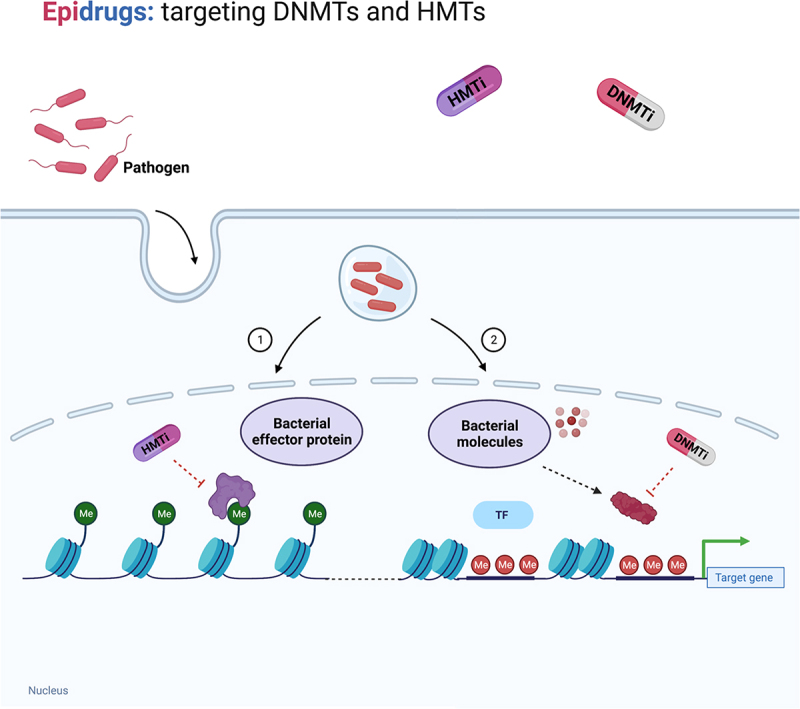
Potential of epidrugs targeting methyltransferases in infection diseases. Targeting methyltransferases in infections could potentially inhibit the epigenetic modifications induced by bacteria in the host. Bacteria release effector proteins, bacterial methyltransferases, (1) or hijack host methyltransferases via release of bacterial molecules and structures (2) to modify the host epigenome. A novel compound targeting bacterial methyltransferase could constitute a novel therapeutic approach. However, this hypothesis needs to be explored. DNA methyltransferase inhibitor (DNMTi), histone methyltransferase inhibitor (HMTi), transcription factor (TF). Created with BioRender (biorender.com).

As described above, bacteria induce DNA and histone methylation modifications upon infection [102]. These chemical modifications of DNA and histone are reversible and chemical probes and drugs have been designed to inhibit these modifications in cancer cells. However, the chemical targeting of the methylation induced by bacteria infection in host cells is still little studied. Due to extensive and improper use of antibiotics and consecutive increase in antibiotic resistance, there is an emerging need for alternative therapies to detain growing antimicrobial resistance (AMR) [103]. Thus, it is of importance to explore alternative therapeutic strategies. The epigenetic alterations induced in the host cells during host-pathogen interactions could be a potential novel target. Specific chemical inhibitors could contribute to elucidate the role of these epigenetic changes and their potential as antibiotics target. Figure 4 depicts a proposed strategy of chemical targeting of DNMTs and HMTs in bacterial infections (Figure 4).

Epigenetic modifications are reversible and thus constitute a potential target. While very little has been explored for bacterial infection, here are several Food and Drug Administration (FDA)-approved epidrugs in the field of cancer [104]. Two drugs are DNA hypomethylation agents, Vidaza® (Azacytidine, 5azaC) and Dacogen® (Decitabine, 5azadC). Both have been approved for the treatment of Myelodysplastic Syndrome (MDS), chronic myelomonocytic leukaemia (CMML) and acute myloid leukaemia (AML) [16,105,106]. Tazverik® (tazemetostat) targets histone methyltransferase EZH2 (responsible for H3K27me2/3) and has been approved for the treatment of epithelioid sarcoma and follicular lymphoma and solid tumours [107–110]. The DOT1L inhibitor named Pinometostat (Epz-5676) was tested in Phase I/II clinical trials for treatment of MLLr leukaemia (clinical trial reference number NCT03701295 and NCT03724084 at https://clinicaltrials.gov), however withdrawn due to poor efficacy. Two PRMT5 inhibitors (GSK3326595 and JNJ-64619178) inhibit the growth of melanoma tumours in murine model and are currently being investigated as the treatment of solid tumours, Non-Hodgkin’s lymphoma and breast cancer [111–113]. The latter are bisusbstrate inhibitors that consist of a chemical moiety mimicking the SAM and one mimicking the substrate linked together with a linker aiming to increase the specificity of the inhibitors [114].

Only one clinical trial study investigating the effect of 5azaC in treatment of pneumonia (clinical trial reference number NCT03941496 at https://clinicaltrials.gov).

Reasoning that upon infection aberrant methylation triggers expression of pro-inflammatory chemokines and cytokines, these aberrant patterns are potential target to reverse inflammation. Indeed, chemical inhibition of methylation diminished release of pro-inflammatory molecules during infection [100]. Zambuzi *et al* showed a decrease in inflammatory cytokines (IL-1β, TNF-α, and IFN-γ) upon treatment with decitabine of mononuclear cells infected with *M. tuberculosis* [115], increasing bacterial phagocytosis and abrogating control of infection. Moreover, treatment with decitabine induced macrophage polarization towards M2 macrophage phenotype. Interestingly, treatment with hypomethylating agent increased bacterial phagocytosis and abrogated control of infection in monocytes [115]. These data support the role of bacterial infections in regulation of inflammation and further studies are needed.

Pathogens also induce changes in the cell environment and immunometabolism that affect the function and enzymatic activity of some methyltransferases. The manipulation of the cell environment may indirectly change the epigenome of the host.

The complexity of the effect of the methylation on cell functions and its role in infection emphasized the importance of studying the epigenetic regulation in host-pathogen interaction.

## Challenges in targeting methyltransferases

The use of epigenetic inhibitors could benefit to the understanding of the molecular mechanism in epigenetic changes upon infection and their development could constitute the starting point for new treatment of infectious diseases. There are several open challenges. The specificity is one. On one hand, all methyltransferases share the same co-factor, the SAM and the catalytic domains are structurally similar (as for example the SET domain of most KMTs) [45]. Interestingly, bacterial effectors can induce directly methylation patterns that are not common in host cells, as H3K14 methylation by *L. pneumophila* [63]. Thus, it is important to design molecules that target specifically bacterial effector proteins without inhibiting host methyltransferases. This could be achieved with bisubstrate inhibitors [114]. On the other hand, all epigenetic methylatransferases are involved in global effects on methylation and are part of protein complexes, interacting with other proteins and epigenetic modifications. Furthermore, targeting epigenetic modifiers should be performed carefully and with consideration of side effects. A recent study of Marcos-Villar *et al* showed that treatment of lung epithelial cells infected with influenza virus with Pinometostat resulted in repression of genes involved in NF-κβ signalling pathway (such as RUBICON and TRIM25), consequently leading to diminished immune response important during infection [116]. These results support further evaluation of the effect of compounds targeting methyltransferases on the expression of genes involved in innate and adaptive immune response in infectious diseases

These are some of the challenges that need to be considered to design efficient inhibitors targeting the epigenetic regulation in bacterial infections.

## Conclusions

In summary, this review emphasizes the important role of DNA and histone methyltransferases in infectious diseases. Bacteria induce changes in DNA and histone methylation pattern in the host by secretion of bacterial effector proteins or hijacking host methyltransferases through bacterial structures and molecules stimuli. Modifications in methylation affect signalling pathways, transcription factors, chromatin structure, host receptors, and microRNAs. This results in altered gene expression involved in immune response, which enables evasion of immune response, bacterial survival, and replication within the host cell (Figure 4). Ultimately, these epigenetic changes contribute to efficient infection. Current studies on compounds that target methyltransferases show clinical relevance in the treatment of diseases where epigenetic patterns are disrupted, for instance in cancer. The studies on epigenetics and inhibitors of methyltransferases in infection could bring a better understanding of the molecular mechanisms of infection. Specific chemical inhibitors could help address these questions. This could contribute to identification of novel treatment strategies that ultimately diminish the use of antibiotics and help to tackle growing antimicrobial resistance. The field is still little explored and more studies are necessary to study the potential and real effect of epidrugs on infection.

## Data Availability

No data was collected for the review, only published and available data were used.

## Abbreviations

5-aza: 5-azacytidine
5-azadC: 5-aza-2’-deoxycytidine
5mC: 5-methylcytosine
DNA: deoxyribonucleic acid
DNMT: DNA methyltransferase
H3: histone 3
H4: histone 4
IL: interleukin
K: lysine
KMT: lysine methyltransferase
LPS: lipopolisascharide
LTA: lipoteichoic acid
MBD: methyl-binding protein
me1: monomethylation
me2: dimethylation
me3: trimethylation
NLS: nucleus localization signal
NF-KB: nuclear factor κβ
PAMP: pathogen associated molecular patterns
PGN: peptidoglycan
PRMT: arginine methyltransferase
R: arginine
SAM: *S*-adenosyl-l-methionine
SAH: *S*-adenosyl-l-homocysteine
TET: ten-eleven translocation dioxygenase
TLR: Toll-like receptor
TNF: tumour necrosis factor-α

## Comment

Figures were created with BioRender (biorender.com).

## References

[1] Watson JD, Crick FH. Molecular structure of nucleic acids; a structure for deoxyribose nucleic acid. Nature. 1953;171(4356):737–16. doi: 10.1038/171737a0 13054692

[2] Piovesan A, Pelleri MC, Antonaros F, et al. On the length, weight and GC content of the human genome. BMC Res Notes. 2019;12(1):106. doi: 10.1186/s13104-019-4137-z 30813969 PMC6391780

[3] Spector DL. Macromolecular domains within the cell nucleus. Annu Rev Cell Biol. 1993;9(1):265–315. doi: 10.1146/annurev.cb.09.110193.001405 8280462

[4] McGinty RK, Tan S. Nucleosome structure and function. Chem Rev. 2015;115(6):2255–2273. doi: 10.1021/cr500373h 25495456 PMC4378457

[5] Borland L, Harauz G, Bahr G, et al. Packing of the 30 nm chromatin fiber in the human metaphase chromosome. Chromosoma. 1988;97(2):159–163. doi: 10.1007/BF00327373 3229179

[6] Zhao S, Allis CD, Wang GG, et al. The language of chromatin modification in human cancers. Nat Rev Cancer. 2021;21(7):413–430. doi: 10.1038/s41568-021-00357-x 34002060 PMC10507815

[7] Lee DS, Luo C, Zhou J, et al. Simultaneous profiling of 3D genome structure and DNA methylation in single human cells. Nat Methods. 2019;16(10):999–1006. doi: 10.1038/s41592-019-0547-z 31501549 PMC6765423

[8] Lee I, Razaghi R, Gilpatrick T, et al. Simultaneous profiling of chromatin accessibility and methylation on human cell lines with nanopore sequencing. Nat Methods. 2020;17(12):1191–1199. doi: 10.1038/s41592-020-01000-7 33230324 PMC7704922

[9] Aloia L, Di Stefano B, Di Croce L, et al. Polycomb complexes in stem cells and embryonic development. Development. 2013;140(12):2525–2534. doi: 10.1242/dev.091553 23715546

[10] Klemm SL, Shipony Z, Greenleaf WJ, et al. Chromatin accessibility and the regulatory epigenome. Nat Rev Genet. 2019;20(4):207–220. doi: 10.1038/s41576-018-0089-8 30675018

[11] Lande-Diner L, Zhang J, Ben-Porath I, et al. Role of DNA methylation in stable gene repression*. J Biol Chem. 2007;282(16):12194–12200. doi: 10.1074/jbc.M607838200 17311920

[12] Chatterjee R, Vinson C. CpG methylation recruits sequence specific transcription factors essential for tissue specific gene expression. Biochim Biophys Acta. 2012;1819(7):763–770. doi: 10.1016/j.bbagrm.2012.02.014 22387149 PMC3371161

[13] Copeland RA. Protein methyltransferase inhibitors as precision cancer therapeutics: a decade of discovery. Philos Trans R Soc B. 2018;373(1748):20170080. doi: 10.1098/rstb.2017.0080 PMC5915721 29685962

[14] Rice JC, Briggs SD, Ueberheide B, et al. Histone methyltransferases direct different degrees of methylation to define distinct chromatin domains. Mol Cell. 2003;12(6):1591–1598. doi: 10.1016/S1097-2765(03)00479-9 14690610

[15] Gong F, Miller KM. Histone methylation and the DNA damage response. Mutat Res Rev Mutat Res. 2019;780:37–47. doi: 10.1016/j.mrrev.2017.09.003 31395347 PMC6690396

[16] Lopez M, Halby L, Arimondo PB, et al. DNA methyltransferase inhibitors: development and applications. Adv Exp Med Biol. 2016;945:431–473. 27826847 10.1007/978-3-319-43624-1_16

[17] Song J, Teplova M, Ishibe-Murakami S, et al. Structure-based mechanistic insights into DNMT1-mediated maintenance DNA methylation. Science. 2012;335(6069):709–712. doi: 10.1126/science.1214453 22323818 PMC4693633

[18] Gao L, Emperle M, Guo Y, et al. Comprehensive structure-function characterization of DNMT3B and DNMT3A reveals distinctive de novo DNA methylation mechanisms. Nat Commun. 2020;11(1):3355. doi: 10.1038/s41467-020-17109-4 32620778 PMC7335073

[19] Feng Q, Wang H, Ng HH, et al. Methylation of H3-lysine 79 is mediated by a new family of HMTases without a SET domain. Curr Biol. 2002;12(12):1052–1058. doi: 10.1016/S0960-9822(02)00901-6 12123582

[20] Li W, Tian W, Yuan G, et al. Molecular basis of nucleosomal H3K36 methylation by NSD methyltransferases. Nature. 2021;590(7846):498–503. doi: 10.1038/s41586-020-03069-8 33361816 PMC7889650

[21] Xu Q, Xiang Y, Wang Q, et al. SETD2 regulates the maternal epigenome, genomic imprinting and embryonic development. Nature Genet. 2019;51(5):844–856. doi: 10.1038/s41588-019-0398-7 31040401

[22] Meng TG, Zhou Q, Ma X-S, et al. PRC2 and EHMT1 regulate H3K27me2 and H3K27me3 establishment across the zygote genome. Nat Commun. 2020;11(1):6354. doi: 10.1038/s41467-020-20242-9 33311485 PMC7733509

[23] Cao R, Wang L, Wang H, et al. Role of histone H3 lysine 27 methylation in Polycomb-group silencing. Science. 2002;298(5595):1039–1043. doi: 10.1126/science.1076997 12351676

[24] Barski A, Cuddapah S, Cui K, et al. High-resolution profiling of histone methylations in the human genome. Cell. 2007;129(4):823–837. doi: 10.1016/j.cell.2007.05.009 17512414

[25] Pinheiro I, Margueron R, Shukeir N, et al. Prdm3 and Prdm16 are H3K9me1 methyltransferases required for mammalian heterochromatin integrity. Cell. 2012;150(5):948–960. doi: 10.1016/j.cell.2012.06.048 22939622

[26] Wang C, Liu X, Gao Y, et al. Reprogramming of H3K9me3-dependent heterochromatin during mammalian embryo development. Nat Cell Biol. 2018;20(5):620–631. doi: 10.1038/s41556-018-0093-4 29686265

[27] Schultz DC, Ayyanathan K, Negorev D, et al. SETDB1: a novel KAP-1-associated histone H3, lysine 9-specific methyltransferase that contributes to HP1-mediated silencing of euchromatic genes by KRAB zinc-finger proteins. Genes Dev. 2002;16(8):919–932. doi: 10.1101/gad.973302 11959841 PMC152359

[28] Falandry C, Fourel G, Galy V, et al. CLLD8/KMT1F is a lysine methyltransferase that is important for chromosome segregation. J Biol Chem. 2010;285(26):20234–20241. doi: 10.1074/jbc.M109.052399 20404330 PMC2888436

[29] Dehé PM, Dichtl B, Schaft D, et al. Protein interactions within the Set1 complex and their roles in the regulation of histone 3 lysine 4 methylation. J Biol Chem. 2006;281(46):35404–35412. doi: 10.1074/jbc.M603099200 16921172

[30] Santos-Rosa H, Schneider R, Bannister AJ, et al. Active genes are tri-methylated at K4 of histone H3. Nature. 2002;419(6905):407–411. doi: 10.1038/nature01080 12353038

[31] Ruthenburg AJ, Allis CD, Wysocka J, et al. Methylation of lysine 4 on histone H3: intricacy of writing and reading a single epigenetic mark. Mol Cell. 2007;25(1):15–30. doi: 10.1016/j.molcel.2006.12.014 17218268

[32] Hamamoto R, Furukawa Y, Morita M, et al. SMYD3 encodes a histone methyltransferase involved in the proliferation of cancer cells. Nat Cell Biol. 2004;6(8):731–740. doi: 10.1038/ncb1151 15235609

[33] Jacques SL, Aquino KP, Gureasko J, et al. CARM1 preferentially methylates H3R17 over H3R26 through a random kinetic mechanism. Biochemistry. 2016;55(11):1635–1644. doi: 10.1021/acs.biochem.5b01071 26848779

[34] Kirmizis A, Santos-Rosa H, Penkett CJ, et al. Distinct transcriptional outputs associated with mono- and dimethylated histone H3 arginine 2. Nat Struct Mol Biol. 2009;16(4):449–451. doi: 10.1038/nsmb.1569 19270702 PMC3350867

[35] Yan Z, Wu H, Liu H, et al. The protein arginine methyltransferase PRMT1 promotes TBK1 activation through asymmetric arginine methylation. Cell Rep. 2021;36(12):109731. doi: 10.1016/j.celrep.2021.109731 34551290

[36] Kim S, Kim NH, Park JE, et al. PRMT6-mediated H3R2me2a guides Aurora B to chromosome arms for proper chromosome segregation. Nat Commun. 2020;11(1):612. doi: 10.1038/s41467-020-14511-w 32001712 PMC6992762

[37] Hu Y, Su Y, He Y, et al. Arginine methyltransferase PRMT3 promote tumorigenesis through regulating c-MYC stabilization in colorectal cancer. Gene. 2021;791:145718. doi: 10.1016/j.gene.2021.145718 33991650

[38] Migliori V, Müller J, Phalke S, et al. Symmetric dimethylation of H3R2 is a newly identified histone mark that supports euchromatin maintenance. Nat Struct Mol Biol. 2012;19(2):136–144. doi: 10.1038/nsmb.2209 22231400

[39] Shoaib M, Chen Q, Shi X, et al. Histone H4 lysine 20 mono-methylation directly facilitates chromatin openness and promotes transcription of housekeeping genes. Nat Commun. 2021;12(1):4800. doi: 10.1038/s41467-021-25051-2 34417450 PMC8379281

[40] Nelson DM, Jaber-Hijazi F, Cole JJ, et al. Mapping H4K20me3 onto the chromatin landscape of senescent cells indicates a function in control of cell senescence and tumor suppression through preservation of genetic and epigenetic stability. Genome Biol. 2016;17(1):158. doi: 10.1186/s13059-016-1017-x 27457071 PMC4960804

[41] Kurup JT, Han Z, Jin W, et al. H4k20me3 methyltransferase SUV420H2 shapes the chromatin landscape of pluripotent embryonic stem cells. Development. 2020;147(23): doi: 10.1242/dev.188516 PMC7725612 33144397

[42] Mikkelsen TS, Ku M, Jaffe DB, et al. Genome-wide maps of chromatin state in pluripotent and lineage-committed cells. Nature. 2007;448(7153):553–560. doi: 10.1038/nature06008 17603471 PMC2921165

[43] Allis CD, Jenuwein T. The molecular hallmarks of epigenetic control. Nat Rev Genet. 2016;17(8):487–500. doi: 10.1038/nrg.2016.59 27346641

[44] Hyun K, Jeon J, Park K, et al. Writing, erasing and reading histone lysine methylations. Exp Mol Med. 2017;49(4):e324–e324. doi: 10.1038/emm.2017.11 28450737 PMC6130214

[45] Dillon SC, Zhang X, Trievel RC, et al. The SET-domain protein superfamily: protein lysine methyltransferases. Genome Bio. 2005;6(8):227. doi: 10.1186/gb-2005-6-8-227 16086857 PMC1273623

[46] Sawada K, Yang Z, Horton JR, et al. Structure of the conserved core of the yeast Dot1p, a nucleosomal histone H3 lysine 79 methyltransferase. J Biol Chem. 2004;279(41):43296–43306. doi: 10.1074/jbc.M405902200 15292170 PMC2688786

[47] Cheng X, Collins RE, Zhang X, et al. Structural and sequence motifs of protein (histone) methylation enzymes. Annu Rev Biophys Biomol Struct. 2005;34(1):267–294. doi: 10.1146/annurev.biophys.34.040204.144452 15869391 PMC2733851

[48] Wesche J, Kühn S, Kessler BM, et al. Protein arginine methylation: a prominent modification and its demethylation. Cell Mol Life Sci. 2017;74(18):3305–3315. doi: 10.1007/s00018-017-2515-z 28364192 PMC11107486

[49] Bussière FI, Michel V, Fernandes J, et al. DNA hypermethylation downregulates Telomerase Reverse Transcriptase (TERT) during H. pylori-induced chronic inflammation. J Oncol. 2019;2019:1–13. doi: 10.1155/2019/5415761 PMC7012206 32082377

[50] Wu X, Zhang Y. TET-mediated active DNA demethylation: mechanism, function and beyond. Nat Rev Genet. 2017;18(9):517–534. doi: 10.1038/nrg.2017.33 28555658

[51] Ginder GD, Williams DC Jr. Readers of DNA methylation, the MBD family as potential therapeutic targets. Pharmacol Ther. 2018;184:98–111. doi: 10.1016/j.pharmthera.2017.11.002 29128342 PMC6438182

[52] Rausch C, Hastert FD, Cardoso MC, et al. DNA modification readers and writers and their interplay. J Mol Biol. 2019;432(6):1731–1746. doi: 10.1016/j.jmb.2019.12.018 31866298

[53] Park I-G, Jeon M, Kim H, et al. Coordinated methyl readers: functional communications in cancer. Semin Cancer Biol. 2021;83:88–99. doi: 10.1016/j.semcancer.2021.03.015 33753223

[54] Gayatri S, Bedford MT. Readers of histone methylarginine marks. Biochim Biophys Acta Gene Regul Mech. 2014;1839(8):702–710. doi: 10.1016/j.bbagrm.2014.02.015 PMC4099268 24583552

[55] Musselman CA, Khorasanizadeh S, Kutateladze TG, et al. Towards understanding methyllysine readout. Biochim Biophys Acta Gene Regul Mech. 2014;1839(8):686–693. doi: 10.1016/j.bbagrm.2014.04.001 PMC4453862 24727128

[56] Zhang L, Lu Q, Chang C. Epigenetics in health and disease. Adv Exp Med Biol. 2020;1253:3–55. 32445090 10.1007/978-981-15-3449-2_1

[57] Sánchez-Romero MA, Casadesús J. The bacterial epigenome. Nat Rev Microbiol. 2020;18(1):7–20. doi: 10.1038/s41579-019-0286-2 31728064

[58] Low DA, Weyand NJ, Mahan MJ, et al. Roles of DNA adenine methylation in regulating bacterial gene expression and virulence. Infect Immun. 2001;69(12):7197–7204. doi: 10.1128/IAI.69.12.7197-7204.2001 11705888 PMC98802

[59] Hattman S, Brooks JE, Masurekar M, et al. Sequence specificity of the P1 modification methylase (M·eco P1) and the DNA methylase (M·eco dam) controlled by the Escherichia coli dam gene. J Mol Biol. 1978;126(3):367–380. doi: 10.1016/0022-2836(78)90046-3 370402

[60] Sakatos A, Babunovic GH, Chase MR, et al. Posttranslational modification of a histone-like protein regulates phenotypic resistance to isoniazid in mycobacteria. Sci Adv. 2018;4(5):eaao1478–eaao1478. doi: 10.1126/sciadv.aao1478 29732401 PMC5931751

[61] Bierne H, Pourpre R. Bacterial factors targeting the nucleus: the growing family of nucleomodulins. Toxins (Basel). 2020;12(4):220. doi: 10.3390/toxins12040220 32244550 PMC7232420

[62] Bierne H, Hamon M, Cossart P, et al. Epigenetics and bacterial infections. Cold Spring Harb Perspect Med. 2012;2(12):a010272. doi: 10.1101/cshperspect.a010272 23209181 PMC3543073

[63] Rolando M, Sanulli S, Rusniok C, et al. Legionella pneumophila effector RomA uniquely modifies host chromatin to repress gene expression and promote intracellular bacterial replication. Cell Host Microbe. 2013;13(4):395–405. doi: 10.1016/j.chom.2013.03.004 23601102

[64] Denzer L, Schroten H, Schwerk C, et al. From gene to protein—how bacterial virulence factors manipulate host gene expression during infection. Int J Mol Sci. 2020;21(10):3730. doi: 10.3390/ijms21103730 32466312 PMC7279228

[65] Schuhmacher MK, Rolando M, Bröhm A, et al. The legionella pneumophila methyltransferase roma methylates also non-histone proteins during infection. J Mol Biol. 2018;430(13):1912–1925. doi: 10.1016/j.jmb.2018.04.032 29733858

[66] Li T, Lu Q, Wang G, et al. SET-domain bacterial effectors target heterochromatin protein 1 to activate host rDNA transcription. EMBO Rep. 2013;14(8):733–740. doi: 10.1038/embor.2013.86 23797873 PMC3736128

[67] Pennini ME, Perrinet S, Dautry-Varsat A, et al. Histone methylation by NUE, a novel nuclear effector of the intracellular pathogen Chlamydia trachomatis. PLOS Pathog. 2010;6(7):e1000995. doi: 10.1371/journal.ppat.1000995 20657819 PMC2904774

[68] Mujtaba S, Winer BY, Jaganathan A, et al. Anthrax SET protein: a potential virulence determinant that epigenetically represses NF-κB activation in infected macrophages. J Biol Chem. 2013;288(32):23458–23472. doi: 10.1074/jbc.M113.467696 23720780 PMC5395026

[69] Yaseen I, Kaur P, Nandicoori VK, et al. Mycobacteria modulate host epigenetic machinery by Rv1988 methylation of a non-tail arginine of histone H3. Nat Commun. 2015;6(1):8922. doi: 10.1038/ncomms9922 26568365

[70] Chernov AV, Reyes L, Xu Z, et al. Mycoplasma CG- and GATC-specific DNA methyltransferases selectively and efficiently methylate the host genome and alter the epigenetic landscape in human cells. Epigenetics. 2015;10(4):303–318. doi: 10.1080/15592294.2015.1020000 25695131 PMC4623497

[71] Lee JC, Kim DS, Moon DC, et al. Prediction of bacterial proteins carrying a nuclear localization signal and nuclear targeting of HsdM from Klebsiella pneumoniae. J Microbiol. 2009;47(5):641–645. doi: 10.1007/s12275-009-0217-4 19851738

[72] Rana S, Maurya S, Mohapatra G, et al. Activation of epigenetic regulator KDM6B by Salmonella Typhimurium enables chronic infections. Gut Microbes. 2021;13(1):1986665. doi: 10.1080/19490976.2021.1986665 34696686 PMC8555538

[73] Manzur KL, Zhou MM. An archaeal SET domain protein exhibits distinct lysine methyltransferase activity towards DNA-associated protein MC1-alpha. FEBS Lett. 2005;579(17):3859–3865. doi: 10.1016/j.febslet.2005.05.026 15978576

[74] Thursby E, Juge N. Introduction to the human gut microbiota. Biochem J. 2017;474(11):1823–1836. doi: 10.1042/BCJ20160510 28512250 PMC5433529

[75] Sztein MB, Bafford AC, Salerno-Goncalves R, et al. Salmonella enterica serovar Typhi exposure elicits ex vivo cell-type-specific epigenetic changes in human gut cells. Sci Rep. 2020;10(1):13581. doi: 10.1038/s41598-020-70492-2 32788681 PMC7423951

[76] Allen J, Hao S, Sears CL, et al. Epigenetic changes induced by bacteroides fragilis toxin. Infect Immun. 2019;87(6):e00447–18. doi: 10.1128/IAI.00447-18 30885929 PMC6529667

[77] Ratajewski M, Walczak-Drzewiecka A, Sałkowska A, et al. Upstream stimulating factors regulate the expression of RORγT in human lymphocytes. J Immunol. 2012;189(6):3034–3042. doi: 10.4049/jimmunol.1200519 22891280

[78] Hock TD, NICK H, AGARWAL A, et al. Upstream stimulatory factors, USF1 and USF2, bind to the human haem oxygenase-1 proximal promoter in vivo and regulate its transcription. Biochem J. 2004;383(2):209–218. doi: 10.1042/BJ20040794 15242350 PMC1134061

[79] Chi TF, Khoder-Agha F, Mennerich D, et al. Loss of USF2 promotes proliferation, migration and mitophagy in a redox-dependent manner. Redox Biol. 2020;37:101750–101750. doi: 10.1016/j.redox.2020.101750 33059314 PMC7566946

[80] Bussière FI, Michel V, Mémet S, et al. H. pylori-induced promoter hypermethylation downregulates USF1 and USF2 transcription factor gene expression. Cell Microbiol. 2010;12(8):1124–1133. doi: 10.1111/j.1462-5822.2010.01457.x 20180799

[81] Mogensen TH. Pathogen recognition and inflammatory signaling in innate immune defenses. Clinical Microbiology Reviews. 2009;22(2):240–273. doi: 10.1128/CMR.00046-08 19366914 PMC2668232

[82] Cole J, Angyal A, Emes RD, et al. Pneumolysin is responsible for differential gene expression and modifications in the epigenetic landscape of primary monocyte derived macrophages. Front Immunol. 2021;12:573266. doi: 10.3389/fimmu.2021.573266 34046027 PMC8145618

[83] Laird MHW, Rhee SH, Perkins DJ, et al. TLR4/MyD88/PI3K interactions regulate TLR4 signaling. J Leukoc Biol. 2009;85(6):966–977. doi: 10.1189/jlb.1208763 19289601 PMC2698589

[84] Stender JD, Pascual G, Liu W, et al. Control of proinflammatory gene programs by regulated trimethylation and demethylation of histone H4K20. Mol Cell. 2012;48(1):28–38. doi: 10.1016/j.molcel.2012.07.020 22921934 PMC3472359

[85] Das A, Chai JC, Kim SH, et al. Transcriptome sequencing of microglial cells stimulated with TLR3 and TLR4 ligands. BMC Genomics. 2015;16(1):517. doi: 10.1186/s12864-015-1728-5 26159724 PMC4497376

[86] de Camargo Pereira G, Guimarães GN, Planello AC, et al. Porphyromonas gingivalis LPS stimulation downregulates DNMT1, DNMT3a, and JMJD3 gene expression levels in human HaCaT keratinocytes. Clin Oral Investig. 2013;17(4):1279–1285. doi: 10.1007/s00784-012-0816-z 22875665

[87] Zhang R, Cheung CY, Seo S-U, et al. RUVBL1/2 complex regulates pro-inflammatory responses in macrophages via regulating histone H3K4 trimethylation. Front Immunol. 2021;12:679184. doi: 10.3389/fimmu.2021.679184 34276666 PMC8282052

[88] Tsai KD, Lee W-X, Chen W, et al. Upregulation of PRMT6 by LPS suppresses Klotho expression through interaction with NF-κB in glomerular mesangial cells. J Cell Biochem. 2018;119(4):3404–3416. doi: 10.1002/jcb.26511 29131380

[89] Timár CI, Lőrincz ÁM, Csépányi-Kömi R, et al. Antibacterial effect of microvesicles released from human neutrophilic granulocytes. Blood. 2013;121(3):510–518. doi: 10.1182/blood-2012-05-431114 23144171 PMC3548170

[90] Sahr T, Escoll P, Rusniok C, et al. Translocated Legionella pneumophila small RNAs mimic eukaryotic microRnas targeting the host immune response. Nat Commun. 2022;13(1):762. doi: 10.1038/s41467-022-28454-x 35140216 PMC8828724

[91] Vdovikova S, Gilfillan S, Wang S, et al. Modulation of gene transcription and epigenetics of colon carcinoma cells by bacterial membrane vesicles. Sci Rep. 2018;8(1):7434. doi: 10.1038/s41598-018-25308-9 29743643 PMC5943334

[92] Armstrong DA, Lee MK, Hazlett HF, et al. Extracellular vesicles from pseudomonas aeruginosa suppress MHC-Related molecules in human lung macrophages. Immunohorizons. 2020;4(8):508–519. doi: 10.4049/immunohorizons.2000026 32819967 PMC7728166

[93] Kyung Lee M, Armstrong DA, Hazlett HF, et al. Exposure to extracellular vesicles from Pseudomonas aeruginosa result in loss of DNA methylation at enhancer and DNase hypersensitive site regions in lung macrophages. Epigenetics. 2021;16(11):1187–1200. doi: 10.1080/15592294.2020.1853318 33380271 PMC8813072

[94] Fonseca S, Carvalho AL, Miquel-Clopés A, et al. Extracellular vesicles produced by the human gut commensal bacterium Bacteroides thetaiotaomicron elicit anti-inflammatory responses from innate immune cells. Front Microbiol. 2022;13:1050271. doi: 10.3389/fmicb.2022.1050271 36439842 PMC9684339

[95] Zhao G, Jones MK, Ottemann KM. Role of bacterial extracellular vesicles in manipulating infection. Infect Immun. 2023;91(5):e0043922. doi: 10.1128/iai.00439-22 37097158 PMC10187128

[96] Schwechheimer C, Kuehn MJ. Outer-membrane vesicles from Gram-negative bacteria: biogenesis and functions. Nat Rev Microbiol. 2015;13(10):605–619. doi: 10.1038/nrmicro3525 26373371 PMC5308417

[97] de Laval B, Maurizio J, Kandalla PK, et al. C/EBPβ-dependent epigenetic memory induces trained immunity in hematopoietic stem cells. Cell Stem Cell. 2020;26(5):657–674.e8. doi: 10.1016/j.stem.2020.01.017 32169166

[98] Moorlag S, Khan N, Novakovic B, et al. β-Glucan Induces Protective Trained Immunity against Mycobacterium tuberculosis Infection: A Key Role for IL-1. Cell Rep. 2020;31(7):107634. doi: 10.1016/j.celrep.2020.107634 32433977 PMC7242907

[99] Arts RJW, Moorlag SJCFM, Novakovic B, et al. BCG vaccination protects against experimental viral infection in humans through the induction of cytokines associated with trained immunity. Cell Host Microbe. 2018;23(1):89–100.e5. doi: 10.1016/j.chom.2017.12.010 29324233

[100] Rasid O, Chevalier C, Camarasa TM-N, et al. H3k4me1 supports memory-like NK cells induced by systemic inflammation. Cell Rep. 2019;29(12):3933–3945.e3. doi: 10.1016/j.celrep.2019.11.043 31851924

[101] Sasaki K, Doi S, Nakashima A, et al. Inhibition of SET domain–containing lysine methyltransferase 7/9 ameliorates renal fibrosis. J Am Soc Nephrol. 2016;27(1):203–215. doi: 10.1681/ASN.2014090850 26045091 PMC4696564

[102] Crimi E, Benincasa G, Cirri S, et al. Clinical epigenetics and multidrug-resistant bacterial infections: host remodelling in critical illness. Epigenetics. 2020;15(10):1021–1034. doi: 10.1080/15592294.2020.1748918 32290755 PMC7518673

[103] Miethke M, Pieroni M, Weber T, et al. Towards the sustainable discovery and development of new antibiotics. Nat Rev Chem. 2021;5(10):726–749. doi: 10.1038/s41570-021-00313-1 PMC8374425 34426795

[104] Rugo HS, Jacobs I, Sharma S, et al. The promise for histone methyltransferase inhibitors for epigenetic therapy in clinical oncology: a narrative review. Adv Ther. 2020;37(7):3059–3082. doi: 10.1007/s12325-020-01379-x 32445185 PMC7467409

[105] Garcia-Manero G, Santini V, Almeida A, et al. Phase III, randomized, placebo-controlled trial of CC-486 (Oral Azacitidine) in patients with lower-risk myelodysplastic syndromes. J Clin Oncol. 2021;39(13):1426–1436. doi: 10.1200/JCO.20.02619 33764805 PMC8099416

[106] Kantarjian H, Issa J-PJ, Rosenfeld CS, et al. Decitabine improves patient outcomes in myelodysplastic syndromes: results of a phase III randomized study. Cancer. 2006;106(8):1794–1803. doi: 10.1002/cncr.21792 16532500

[107] Italiano A, Soria J-C, Toulmonde M, et al. Tazemetostat, an EZH2 inhibitor, in relapsed or refractory B-cell non-Hodgkin lymphoma and advanced solid tumours: a first-in-human, open-label, phase 1 study. Lancet Oncol. 2018;19(5):649–659. doi: 10.1016/S1470-2045(18)30145-1 29650362

[108] Morschhauser F, Tilly H, Chaidos A, et al. Tazemetostat for patients with relapsed or refractory follicular lymphoma: an open-label, single-arm, multicentre, phase 2 trial. Lancet Oncol. 2020;21(11):1433–1442. doi: 10.1016/S1470-2045(20)30441-1 33035457 PMC8427481

[109] Gounder M, Schöffski P, Jones RL, et al. Tazemetostat in advanced epithelioid sarcoma with loss of INI1/SMARCB1: an international, open-label, phase 2 basket study. Lancet Oncol. 2020;21(11):1423–1432. doi: 10.1016/S1470-2045(20)30451-4 33035459

[110] Straining R, Eighmy W. Tazemetostat: EZH2 Inhibitor. J Adv Pract Oncol. 2022;13(2):158–163. doi: 10.6004/jadpro.2022.13.2.7 35369397 PMC8955562

[111] Li X, Wang C, Jiang H, et al. A patent review of arginine methyltransferase inhibitors (2010–2018). Expert Opin Ther Pat. 2019;29(2):97–114. doi: 10.1080/13543776.2019.1567711 30640571

[112] Kim H, Kim H, Feng Y, et al. PRMT5 control of cGAS/STING and NLRC5 pathways defines melanoma response to antitumor immunity. Sci Transl Med. 2020;12(551): doi: 10.1126/scitranslmed.aaz5683 PMC7508354 32641491

[113] Siu LL, Rasco DW, Vinay SP, et al. 438O - METEOR-1: A phase I study of GSK3326595, a first-in-class protein arginine methyltransferase 5 (PRMT5) inhibitor, in advanced solid tumours. Ann Oncol. 2019;30:v159. doi: 10.1093/annonc/mdz244

[114] Bon C, Halby L, Arimondo PB, et al. Bisubstrate inhibitors: the promise of a selective and potent chemical inhibition of epigenetic ‘writers’. Epigenomics. 2020;12(17):1479–1482. doi: 10.2217/epi-2020-0203 32938211

[115] Zambuzi FA, Cardoso-Silva PM, Castro RC, et al. Decitabine promotes modulation in phenotype and function of monocytes and macrophages that drive immune response regulation. Cells. 2021;10(4):868. doi: 10.3390/cells10040868 33921194 PMC8069756

[116] Marcos-Villar L, Nieto A. The DOT1L inhibitor Pinometostat decreases the host-response against infections: considerations about its use in human therapy. Sci Rep. 2019;9(1):16862. doi: 10.1038/s41598-019-53239-6 31727944 PMC6856118

